# Understanding Mental Health Clinicians’ Perceptions and Concerns Regarding Using Passive Patient-Generated Health Data for Clinical Decision-Making: Qualitative Semistructured Interview Study

**DOI:** 10.2196/47380

**Published:** 2023-08-10

**Authors:** Jodie Nghiem, Daniel A Adler, Deborah Estrin, Cecilia Livesey, Tanzeem Choudhury

**Affiliations:** 1 Medical College Weill Cornell Medicine New York, NY United States; 2 College of Computing and Information Science Cornell Tech New York, NY United States; 3 Optum Labs UnitedHealth Group Minnetonka, MN United States; 4 Department of Psychiatry University of Pennsylvania Philadelphia, PA United States

**Keywords:** digital technology, clinical decision support, mobile health, mHealth, qualitative research, mental health, clinician, perception, patient-generated health data, mobile app, digital app, wearables, mobile phone

## Abstract

**Background:**

Digital health-tracking tools are changing mental health care by giving patients the ability to collect passively measured patient-generated health data (PGHD; ie, data collected from connected devices with little to no patient effort). Although there are existing clinical guidelines for how mental health clinicians should use more traditional, active forms of PGHD for clinical decision-making, there is less clarity on how passive PGHD can be used.

**Objective:**

We conducted a qualitative study to understand mental health clinicians’ perceptions and concerns regarding the use of technology-enabled, passively collected PGHD for clinical decision-making. Our interviews sought to understand participants’ current experiences with and visions for using passive PGHD.

**Methods:**

Mental health clinicians providing outpatient services were recruited to participate in semistructured interviews. Interview recordings were deidentified, transcribed, and qualitatively coded to identify overarching themes.

**Results:**

Overall, 12 mental health clinicians (n=11, 92% psychiatrists and n=1, 8% clinical psychologist) were interviewed. We identified 4 overarching themes. First, passive PGHD are patient driven—we found that current passive PGHD use was patient driven, not clinician driven; participating clinicians only considered passive PGHD for clinical decision-making when patients brought passive data to clinical encounters. The second theme was active versus passive data as subjective versus objective data—participants viewed the contrast between active and passive PGHD as a contrast between interpretive data on patients’ mental health and objective information on behavior. Participants believed that prioritizing passive over self-reported, active PGHD would reduce opportunities for patients to reflect upon their mental health, reducing treatment engagement and raising questions about how passive data can best complement active data for clinical decision-making. Third, passive PGHD must be delivered at appropriate times for action—participants were concerned with the real-time nature of passive PGHD; they believed that it would be infeasible to use passive PGHD for real-time patient monitoring outside clinical encounters and more feasible to use passive PGHD during clinical encounters when clinicians can make treatment decisions. The fourth theme was protecting patient privacy—participating clinicians wanted to protect patient privacy within passive PGHD-sharing programs and discussed opportunities to refine data sharing consent to improve transparency surrounding passive PGHD collection and use.

**Conclusions:**

Although passive PGHD has the potential to enable more contextualized measurement, this study highlights the need for building and disseminating an evidence base describing how and when passive measures should be used for clinical decision-making. This evidence base should clarify how to use passive data alongside more traditional forms of active PGHD, when clinicians should view passive PGHD to make treatment decisions, and how to protect patient privacy within passive data–sharing programs. Clear evidence would more effectively support the uptake and effective use of these novel tools for both patients and their clinicians.

## Introduction

### Background

Digital health-tracking technologies continue to gain popularity. The adoption of these tools is steadily increasing, fueled by recent innovations in smartphone and wearable technologies that allow for the collection and storage of health-related data. Patients use the data collected by these consumer technologies to self-monitor their health and well-being [1]. This adoption disrupts how health-related data have been traditionally collected and used—patients are driving adoption without recommendations from clinical providers on what data to collect and how collected data should be interpreted and used for health-related decision-making.

The increasing adoption of digital health tracking has led to a growing interest in using patient-generated health data (PGHD), defined as “health-related data created, recorded, or gathered by or from patients (or family members or other caregivers) to help address a health concern,” for clinical decision-making [2]. PGHD can be categorized by the amount of user participation required for use as health data. *Active* PGHD require user participation during health data collection and have traditionally included health surveys such as patient-reported outcome (PRO) surveys collected via analog (eg, pen and paper surveys) and digital (eg, mobile apps) media, as well as health histories gathered by patients. More novel forms of active PGHD also include behavioral data collected during specific health-related active tasks in partially controlled conditions (eg, keystroke data collected during specific typing tasks) [3].

Recently, clinicians have also considered using *passively* collected PGHD in care. Passive PGHD are data automatically collected by digital platforms (eg, smartphones and websites) from everyday life with little to no user participation or data that exist on these platforms from everyday interactions and can be repurposed for health monitoring and treatment without additional user effort. Examples of passive PGHD include activity data automatically collected by smartphones or wearable devices or data from social media interactions that are repurposed for health care use (Table 1) [4-8]. Both active and passive PGHD can be transformed into digital biomarkers to inform the clinical management of medical conditions, including but not limited to diagnosis, monitoring, and prognosis [9], and clinicians are beginning to use passive PGHD in their clinical practice to treat a wide variety of chronic conditions such as asthma, cancer, and diabetes [4].

**Table 1:** Definitions and examples of passive and active patient-generated health data.

Characteristic	Passive	Active
Definition	Data collected or repurposed for health monitoring and treatment without user participation [5]	Health data requiring user participation for collection [5]
Medium	Digital media, including smartphones, wearables, or web platforms	Analog (eg, pen and paper) or digital media
Setting	Collected from everyday life	Collected during specific tasks to measure or document health-related information
Examples	Step counts and inferred activity collected from a smartphone or wearable device [10] Sleep duration approximated from heart rate and acceleration [11] Mobility information calculated from mobile phone location-based services [12] Data from social media interactions repurposed for health monitoring and treatment [6-8]	Self-reported health outcomes (eg, PHQ-9^a^ and GAD-7^b^) Health histories gathered by patients Data collected from users during active tasks in partially controlled conditions, for example, keystroke data collected from performing specific typing tasks [3] Journaling as an intervention to improve mental health [13]

^a^PHQ-9: Patient Health Questionnaire–9.

^b^GAD-7: Generalized Anxiety Disorder–7.

In recent years, researchers have speculated on how mental and behavioral health care may specifically benefit from collecting and using passive PGHD. Approximately 1 in 5 adults in the United States experience mental illness each year, and those experiencing symptoms have difficulties accessing treatment because of a national shortage of mental health care providers [14]. Passive PGHD may provide clinicians with contextual data on their patients from outside the clinical encounter to improve decision-making between often short, infrequent visits [15,16]. Motivated by this promise and the pressure to vet the many new solutions coming into the market, researchers have investigated the validity of using passive PGHD to measure mental health. For example, smartphones can collect data on behavior and social routines associated with symptoms of affective and mood disorders [17,18]. Data from social media (eg, Facebook, Twitter, and Instagram) add semantically relevant information to provide a more in-depth understanding of patients’ psychosocial behaviors [19,20].

However, even with refined measures, it is unclear how mental health clinicians can use passive PGHD for clinical decision-making. There are limited clinical guidelines regarding the use of passive PGHD in mental health care. Clinical guidelines exist for the use of specific types of active PGHD traditionally used to measure mental health symptoms, specifically PROs. For example, governmental and professional bodies have outlined the appropriate use of the Patient Health Questionnaire–9 and Generalized Anxiety Disorder–7 for depression and anxiety screening, respectively [21-23]. Similar guidelines do not exist for the use of passive PGHD, although companies developing passive PGHD collection technologies such as the Apple Watch and Oura Ring have created instruction manuals [24,25] to guide consumer use. Without clinical guidelines, the safe and effective use of passive PGHD for clinical decision-making is unclear. In this study, we wished to aid clinical guideline development by speaking with mental health clinicians about their perceptions and concerns regarding the use of passive PGHD in clinical care.

### Related Work

Prior work has focused on how PGHD may augment clinical encounters. Wu et al [26] explored mental health clinicians’ perspectives on PGHD and how PGHD fit into current clinical workflows. Ng et al [27] focused specifically on how passive PGHD may be integrated into an intensive treatment program for veterans with posttraumatic stress disorder. This work has primarily sought to understand how PGHD fit into the clinical workflow (an operational process) without considering the broader view of how PGHD may reshape clinical decision-making (a cognitive process) [28,29]. Although PGHD integration can facilitate adoption [26], it does not guarantee that clinicians will ultimately find these data useful for clinical decision-making. In addition, researchers have surfaced ethical tensions surrounding passive PGHD use in clinical care. For example, inherent in the use of passive data are reduced transparency in data collection and the automatic repurposing of nonclinical data for clinical use [5]. We aimed to explore with participants how these concerns shaped their perceptions of using passive PGHD for clinical decision-making.

More recently, Schmidt and D’Alfonso [30] considered with mental health clinicians, mostly psychologists delivering therapy, how *digital phenotyping*, a paradigm in which mental state is inferred from a patient’s digital footprint (including both passive PGHD and digitally collected self-reports), can be used to inform client treatment. In this work, we considered with mental health clinicians, mostly psychiatrists delivering therapy plus other forms of treatment (eg, medication management), the broader potential of passive PGHD in clinical decision-making not only for inferring mental state as implied by the digital phenotyping paradigm.

### Contribution

We contribute with a qualitative study to better understand mental health clinicians’ perceptions and concerns regarding the use of technology-enabled, passively collected PGHD for clinical decision-making. Specifically, we conducted 12 semistructured interviews with mental health clinicians, including 11 (92%) psychiatrists and 1 (8%) clinical psychologist, and performed qualitative coding to derive 4 themes from these interviews (Textbox 1).

**Overarching themes derived from the interviews.**

**Current passive patient-generated health data (PGHD) use is patient driven**

- Participating clinicians’ current experiences with passive PGHD were patient driven, not clinician driven, limited to moments when patients chose to collect passive PGHD and bring these data to clinical encounters.

**Active versus passive data as subjective versus objective data**

- Participating clinicians viewed contrasts between active and passive PGHD as a contrast between subjective, interpretive data on patients’ mental health, specifically referring to self-reports and health histories traditionally used to measure mental health symptoms, and objective information on behavior but not necessarily mental health. Participants believed that prioritizing passive over active self-reports would reduce opportunities for patients to reflect on their mental health and change their engagement in treatment, raising questions about how passive data can complement active data for clinical decision-making.

**Passive PGHD must be delivered at appropriate times for action**

- Our participants were concerned with the real-time nature of passive PGHD—within current workflows, participants believed that it would be infeasible to use passive PGHD for real-time patient monitoring outside clinical encounters and more feasible to use passive PGHD at moments surrounding clinical encounters when they can make treatment decisions.

**Protecting patient privacy**

- Participating clinicians wanted to protect patient privacy within passive PGHD-sharing programs and discussed opportunities to refine data sharing consent to improve transparency surrounding passive PGHD collection and use.

## Methods

### Study Overview

We conducted semistructured interviews with mental health clinicians. These interviews collected qualitative data on clinicians’ current understanding and use of PGHD as well as how clinicians could use PGHD for clinical decision-making in the future. Methodologically, we were motivated by the speculative design methods referenced by Malpass [31] to open up a conversation with participants on how their current practices may contribute to a future in which passive PGHD are used for clinical decision-making. Similar to the notion of speculative design that Malpass [31] describes, our objective was not to design any specific technology but to understand how participants would shape a future where passive PGHD is used for clinical decision-making. We were also motivated by the idea of speculative data introduced by Hockenhull and Cohn [32] to uncover how data, specifically passive PGHD, may be produced, validated, and used by participants in the future. In this section, we detail our methodology, including the study procedures and methods for analysis.

### Interview Design

We created an interview guide composed of 3 sections following the speculative design tradition [31,33]. The interview opened with an introductory section to learn about the participants’ clinical practice. We then explored participants’ current understanding of PGHD by asking them about the forms of PGHD they used and how they used them in clinical decision-making. This section included further questions for participants who were less familiar with PGHD to establish a baseline along the full spectrum of familiarity with PGHD. Finally, we explored participants’ future interests in PGHD by asking them about the forms of PGHD that they would choose to use in clinical care and how these choices could affect decision-making. Although participants were approached as clinicians, the semistructured, open-ended nature of the interview guide was designed to provide participants with the opportunity to elaborate upon how any experiences outside the clinical space might influence their attitudes toward PGHD. Please see Multimedia Appendix 1 for the full interview guide.

### Participant Recruitment

We enrolled mental health clinicians as participants, including but not limited to psychiatrists, clinical psychologists, and licensed clinical social workers delivering outpatient services in the New York City Metro Area in the United States. Our study focused on mental health clinicians delivering outpatient services as passive PGHD offer particular promise for understanding patients’ activities in between outpatient visits [15,16]. Participants were recruited using both convenience and purposive sampling and were assured that their participation would not affect their relationship with their employer or our research institution [34].

We sought to conduct in-depth one-on-one interviews allowing for question adaptation and probes to reveal insights that could not be obtained from surveys. This required us to be respectful of participants’ time, not further burdening them to engage in this research. Mental health clinicians face many emotional burdens daily, and their bandwidth was reduced during our recruitment period in early 2022 with the ongoing COVID-19 pandemic exacerbating mental health concerns [35]. Given the nature of our work and the pressures our study population faced at the time of these interviews, we spoke with 12 mental health clinicians. The self-reported demographic information of our participants is summarized in Table 2.

**Table 2:** Self-reported demographic information of the study participants (N=12).

Characteristic		Values
Age (years)^a^, mean (SD)		50 (17)
Female^a^, n (%)		5 (42)
**Specialty (degree), n (%)**		
	Psychiatry (MD^b^)	11 (92)
	Psychology (PhD^c^)	1 (8)
**Years practicing, n (%)**		
	≤10	5 (42)
	11-20	2 (17)
	21-30	2 (17)
	>30	3 (25)
**Practice setting, n (%)**		
	Private practice	6 (50)
	Academic medical center	5 (42)
	Employee assistance programs	1 (8)

^a^One participant preferred not to disclose their age or gender.

^b^MD: Doctor of Medicine.

^c^PhD: Doctor of Philosophy.

### Data Collection and Analysis

Interviews were held via 35-minute Zoom (Zoom Video Communications) meetings and were conducted by the 2 co–first authors. Study sessions were audio recorded, transcribed using a professional service, and anonymized. The 2 co–first authors analyzed the anonymized transcripts using a thematic analysis approach [36-38]. Guided by the literature and research objectives, the transcripts were independently open coded. The coded transcripts were then reviewed to reach a joint interpretation and agreement toward a final codebook. Final codes were qualitatively clustered into the 4 themes based on the meaning detailed in the *Results* section. Specific codes and high-level themes can be found in Multimedia Appendix 2. Examples of codes included *design and functionality of PGHD solutions*, *validity of passive measures*, *patient-reported outcomes as PGHD*, and *PGHD and the therapeutic frame*.

### Ethics Approval

The study procedures were approved by the Institutional Review Board for Human Participant Research at Cornell University (protocol number IRB0010706).

### Informed Consent and Participation

Participants were asked to provide informed consent after receiving a complete description of the study. Eligible participants had the option of not providing consent and could withdraw from the study at any point. Data collected during the interviews (transcripts and notes taken during the interviews) were deidentified.

### Researcher Positionality

One of the 2 co–first authors is a medical student at an academic medical center located in New York City, and the second co–first author is a graduate student in computer and information science. These authors recruited participants, conducted semistructured interviews, and analyzed the transcripts. All other authors contributed to drafting and revising the manuscript and did not see the data.

## Results

### Overview

Our results are summarized within 4 themes illuminating participating mental health clinicians’ perceptions and concerns regarding the use of passive PGHD as well as participants’ speculation on how passive PGHD could be used for clinical decision-making. These themes and findings are summarized in Textbox 2. Participants are quoted throughout our results using a participant-specific identification number (eg, P12) to retain anonymity.

**Summary of themes and findings.**

**Current passive patient-generated health data (PGHD) use is patient driven**

- Participants’ current experiences with passive PGHD were limited to times when patients collected passive data and brought these data into clinical encounters.
- Participants chose to not use passive PGHD as they believed that passive PGHD only measured a limited set of behaviors related to mental health and questioned the accuracy of these measures compared with measurements taken during clinical behavioral studies (eg, sleep studies).

**Active versus passive data as subjective versus objective data**

- Participants perceived that prioritizing passively collected PGHD in place of active, self-reported data during clinical visits would change patients’ engagement in their treatment by reducing opportunities for patients to reflect on and interpret their mental health.
- Participants were interested in reviewing discrepancies between passive and active PGHD with patients.
- Some participants prioritized collecting active PGHD for specific treatments (eg, mental health history in psychodynamic therapy) pending the patient’s condition.

**Passive PGHD must be delivered at appropriate times for action**

- Participants were worried about the burden of and liability to review real-time passive PGHD outside clinical encounters.
- Participants imagined that this liability could be mitigated by ordering passive PGHD similarly to a lab test. Thus, participants would only be liable to review passive measures when there was a clear clinical justification for use.
- Similarly, real-time passive PGHD could be dosed similar to a prescription, restricted to times when it made sense to hyperfocus on the relationships between behavior and mental health.

**Protecting patient privacy**

- Participants wanted consent for passive PGHD collection to be a guided, hands-on process.
- Incorporating goal setting into the consent process was seen as a potential method to clearly align patients’ goals with PGHD collection.
- Participants hesitant to engage patients in data sharing were concerned that PGHD may disclose information to clinicians that patients would have preferred to keep private, potentially violating the therapeutic frame.

### Current Passive PGHD Use Is Patient Driven

We began to explore participating clinicians’ current experience with passive PGHD by asking them if they had used passive PGHD for clinical decision-making. Participants reported some familiarity with passive PGHD, including *“*step counts or other things that could be picked up either via their phone or otherwise beyond mental health scales” (P2) or *“*a sleep app to look at the number of hours slept and the quality of the sleep” (P1). Despite this familiarity, participants overall did not appear to currently use passive PGHD in their clinical practice. Instead, participants stated that passive PGHD use was patient driven; in other words, they only used passive PGHD in clinical decision-making when patients themselves brought their passive data into the clinical visit. For example, one of our participants mentioned how patients would bring passively collected sleep data to their appointments:

I have a couple of patients who wear WHOOPs [a fitness wearable equipped with sensors to measure physiologic data] [39]. I don’t have that much familiarity with what those are, but they will sometimes send me their data. And that tends to come up the most when people are trying to quantify issues with sleep... And we talk about, how much has your sleep quality been? ...And they’ll like sending me that data and having me kind of follow along with them. I don’t recommend doing it, but I accept it when patients want to send it to me.

P8

Thus, this participant did not actively recommend that her patients collect these data but did not refuse to review them with her patients if they chose to collect them and bring them into the clinical encounter. Other participants shared similar experiences of patients bringing them their passively collected data. For example, P2 mentioned that they had “patients who’ve done sleep studies who have apps that show their sleep” and that they (the participant) “love that.”

We asked participants why they did not currently use passive PGHD for clinical decision-making and instead only used passive PGHD when patients wanted to review these data with their clinicians. Participants stated that this was because current passive measures only contained information about a limited set of health behaviors. For example, a participant mentioned that they believed that “steps data gets tricky because I think oftentimes people are physically active in other ways” (P6). Another participant questioned whether passive PGHD accurately measured behavior compared with other “gold standard” ways of measuring behavior for clinical use. For example, this participant stated that they “don’t know enough to say that those sleep monitors are tracking your sleep compared with a sleep study with an EEG and everything else” (P5).

In summary, participating clinicians were familiar with passive PGHD. However, participating clinicians’ current experiences with using passive PGHD for clinical decision-making were patient driven, not clinician driven, limited to times when patients chose to collect passive data and review them with their clinicians during clinical encounters. Participants did not currently use passive PGHD for clinical decision-making as they found that existing passive measures only contained information about a limited set of health behaviors (eg, can measure step counts but not all other aspects of physical activity). In addition, participants questioned whether passive PGHD accurately measured behavior compared with clinical studies (eg, sleep studies).

### Active Versus Passive Data as Subjective Versus Objective Data

The previous section highlights that participating clinicians did not choose to use passive PGHD in clinical decision-making. Clinicians in this study were more familiar with certain forms of active PGHD, specifically PROs and health history information traditionally gathered by patients to assess mental health symptoms. Given their familiarity with these forms of active PGHD, we probed participants further to understand their thoughts and preferences regarding how passive and active PGHD could be jointly used for clinical decision-making.

We first compared and contrasted these 2 types of information with participants to understand their perceptions of what each data type offered. Participants drew the contrast between active self-reports and passive data as a contrast between information describing a patient’s *subjective* interpretation of their mental health and more *objective* information on behavior but not necessarily mental health. For example, a participant directly noted this contrast, stating that “a patient reflecting on their mental state is not the same as...hours of sleep, number of steps, their heart rate, things like that” (P2). In addition, this participant was concerned about how prioritizing passively collected PGHD over active data changed the extent of patient participation in measuring symptoms. In particular, the participant noted how passive PGHD may reshape patients’ engagement in symptom measurement and data generation as, when collecting active, self-reported data, “you’re asking them [patients] to reflect on their internal experience,” whereas passive data are “just picking updates that’s certainly about them [the patient], but it feels like they’re less a part of the choice process of, or the generation of that data” (P2).

Participants were also interested in how the 2 types of data (active and passive) may validate or invalidate each other. Although one might expect that such discrepancies would call into question the validity of a patient self-report or passive measure, clinicians in our study were more interested in how these discrepancies could be leveraged within treatment. A participant, an addiction psychiatrist, recalled a time when they used passive PGHD to help change a patient’s perception of how their drug use affected their behavior:

I just had someone check their sleep before and after stopping marijuana. Their hypothesis was that they would sleep much poorer without the weed and it turned out they slept much better, as I anticipated. I told them, “Let’s run this experiment.” I think the benefit comes from them [the patient] drawing their conclusions from their data.

P11

Thus, engaging with discrepancies across data types became an active part of the treatment process. That being said, not all clinicians in our study agreed that focusing on these differences would benefit clinical decision-making. Some of the clinicians we interviewed thought that focusing on passively collected PGHD may make it more difficult to reconcile patients’ subjective experiences with their mental health. For example, a clinician stated that, if passive and active data conflicted, they would be less interested in the “conflicting information unless I’m making a biological change” (P12).

We pressed this clinician to elaborate further on why they might be less interested in the conflicting information. In response, this participant described how the main form of treatment they practiced, psychodynamic therapy, prioritized patients’ interpretation of their lived experience:

I mean, the thing is that a person’s subjective experience is a kind of reality, right? Sometimes objective information is irrelevant because you’re like, “Okay, well I don’t care what that says. What I felt was this.” So subjective information sometimes outweighs objective information. ...What actually happened doesn’t matter. It’s what you remember or what you feel that matters.

P12

Another clinician we interviewed agreed with this sentiment, stating that it does not “really matter what the objective data is” as a patient may self-report “feeling absolutely awful” even though “their objective data, their wearables, look much improved.” Thus, if “they [the patient] doesn’t feel well, how much does it [the objective data] really matter?” (P4). However, this participant did state that deprioritizing what they called objective data would only make sense in specific situations, for example, if “their metrics [passive data] look bad, but they feel great and they’re doing very well and they’re not manic or vice versa” (P4), implying that it is important to contextualize data with the patient’s current condition (ie, mania).

In summary, participants viewed the shift from active self-reported data toward passive data as a shift from subjective, interpretive data on mental health toward more objective information on behavior. Participants were less concerned about comparing the validity of actively versus passively collected PGHD but more interested in how shifting from active to passive data decreases the opportunity for patient reflection, a critical part of mental health care. Clinicians then elaborated on how the complementary, potentially conflicting information from active and passive data could inform treatment by helping patients realize progress and potentially re-engage. Finally, some participants stated that the degree to which they would prioritize active data, highlighting the patient’s subjective interpretation of their mental health, would depend on the patient’s clinical state (ie, “not manic”) as well as the type of intervention being delivered (eg, psychodynamic therapy). We contextualize these discrepancies further in the *Discussion* section.

### Passive PGHD Must Be Delivered at Appropriate Times for Action

The first section highlighted how most participants had limited experience with using passive PGHD for clinical decision-making. Despite this limited experience, the previous section highlights that participants still believed that passive PGHD could complement more traditional forms of active PGHD to improve clinical decision-making. As such, we were interested in exploring how passive PGHD can be delivered within participants’ workflows such that they can act upon passive PGHD for clinical decision-making.

As we began to investigate with participants how to best deliver passive PGHD, a participant imagined a system in which passive PGHD were integrated in real time into the patients’ charts. These data would be available to clinicians outside specific clinical encounters. The real-time potential of passively collected PGHD worried participants, specifically if the data indicated that a patient was in need of immediate care:

Nobody wants to get, “My patient’s suicidal Saturday at 2:00 PM” So it’s got to be done in a very thoughtful way. But if you said your patient’s score that you’re seeing in 30 minutes just changed from a 10 to a 20, that’s helpful. That gives me actionable data at the time I need it to help improve the visit and take better care of them. That I would call useful. But don’t just send me random things at random times.

P2

Participants further stated that it would be next to impossible to expect clinicians to review continuous behavioral data. Participants already experienced data overload during their work, and adding passive data may further contribute to this overload. A participant warned the following:

If you’re going to overload people with things and then they become required, you’re going to be further burning the healthcare workers who are already resigning in droves and burning out very quickly.

P4

Participants were further concerned about the inherent liability of not acting upon real-time data outside the clinical encounter. A participant used an analogy comparing passive data to laboratory tests:

It’s as if you order labs, you have to follow up on those labs. You can’t just wait a month or three months to see those labs. Someone’s got to track those labs.

P4

As laboratory tests are typically ordered within clinical encounters, ordering passive PGHD similarly to a laboratory test would restrict the use of passive data to moments when clinicians have the bandwidth to use passive PGHD for decision-making.

However, it is possible that there could be a clinical justification to collect real-time passive PGHD, potentially to monitor high-risk patients outside clinical encounters. A participant imagined that real-time passive PGHD could be collected for limited periods, essentially dosing the use. By dosing passive PGHD similarly to a prescription, “you’re getting people for a limited period of time to really hyperfocus on the connections between their mood and their activities and their triggers and all that” (P4). Thus, the potential for real-time monitoring through passive PGHD could be reduced to limited periods when there is a clinical justification for continuous monitoring.

Participants also speculated that, without clearly defining a dose for passive PGHD (eg, how frequently to review PGHD and what PGHD to review), the abundance and continuity of passive PGHD may create obsessive tendencies in patients. Many of our participants’ patients already experienced anxiety, which may increase if patients are able to visualize granular, real-time fluctuations in their behavior and health. Participants worried that, if one were “to be [collecting passive data] on a very long term basis might just make people obsessive” (P4). As a participant noted, this risk of obsession is especially relevant in mental health given the interpretive nature of mental health data compared with data collected within other fields of medicine:

It’s not the same as blood pressure, where someone’s like, “I measured my blood pressure and these were my readings.” It’s going to be a little bit more complicated to tell the story, because there are no vital signs in psychiatry. It’s usually a lot more subtle and open for interpretation.

P9

In summary, participants were reluctant to adopt passive PGHD for decision-making without established clinical guidelines that articulate when and how clinicians and their patients should review and interpret these data. Despite these barriers, participants were not entirely dismissive of using passive PGHD. Some participants imagined how established clinical practices, including ordering laboratory tests or prescribing treatments, could be repurposed to most effectively use PGHD for clinical decision-making.

### Protecting Patient Privacy

The previous section highlights that participating clinicians overall were open to discussing how to appropriately integrate PGHD into clinical visits for decision-making. As we probed participants further, many noted the sensitivity of collecting and storing passive PGHD as passive measures offer a magnifying glass into the daily routines and habits of their patients. A participant stated that “It feels weird, intuitively feels weird” to collect passive PGHD*,* and this participant began speculating on who may have access to data in proprietary systems (“the military? ...the government? ...corporations?”), begging the question of “would patients feel comfortable” (P12) sharing their data.

Given this tension, we attempted to navigate with participants different controls to protect patient privacy if passive PGHD were used for clinical decision-making. For example, many participants raised how to best consent patients into PGHD-sharing programs. A participant stated that consent to share PGHD would need to be a guided, hands-on process, not “another, ‘I agree,’ click button,” and that patients “have to actually talk through it with someone” (P2).

We probed this participant to further understand how a patient and their clinician might discuss collecting PGHD. This participant stated that patients would be encouraged to share PGHD if the latter directly aligned with their treatment goals:

I think the best way is to make it explicit that we decided together this is the goal of treatment and that’s why we’re tracking it. ...Can you envision a world in which like in the patient portal, they select their three goals and one of them is exercise? They’re actually signing up for it.

P2

Through this explicit alignment, participants hypothesized that patients would see a direct benefit to their health in sharing PGHD. As a participant noted, *“*if you can show useful outcomes” and “prove to the patient that this is really going to have an impact,” patients “don’t mind sharing their data” (P1).

A participant noted that the process of negotiating sharing PGHD with a patient may actually be a useful part of treatment. This participant discussed how it was often difficult for participants to convey certain types of information during the clinical visit. Sharing PGHD may actually help patients open up about some of these experiences, as this participant stated:

Trauma needs a witness. You’ve got to get the stuff out there in the world if you’re going to be able to do something with it. ...And now people are more willing to talk about it, to share data, to look at it. When you can get to that point of being open and honest about sort of what’s going on inside you or what data you’re presenting, it makes it much easier to change.

P11

Some participants were more hesitant to engage patients in sharing their PGHD. In particular, these participants stated that patients should remain in complete control over disclosing personal information. These participants were concerned that sharing PGHD may lead patients to unintentionally disclose information to their clinicians that they would prefer to keep private. A clinician noted this with regard to the therapeutic relationship:

My goal when I’m wearing my psychoanalytic hat is to see the world through my patient’s eyes. Any other data that I get constitutes an interference with that.

P3

Thus, participants saw data sharing as a delicate balance. On the one hand, sharing PGHD could be a tool to further engage patients in their treatment, creating conversations between patients and clinicians that may be more difficult to motivate without being prompted by the information contained within PGHD. In contrast, participants noted that they would need to be careful about how they engaged patients in their PGHD, having the patient remain in control of what information is or is not discussed. This discourse opens up a fruitful discussion on how to best center patients’ interests within passive PGHD programs.

## Discussion

### Principal Findings

We conducted a qualitative study with mental health clinicians to better understand their perceptions and concerns regarding the use of passively collected PGHD for clinical decision-making. Our results highlight a range of opportunities and challenges that clinicians foresee toward using PGHD in clinical care. Broadly, our participants believed that passive PGHD could be used to improve engagement in some aspects of care, giving patients and their clinicians the ability to set goals and reflect on highly specific contextual data within patients’ everyday lives. These data would create opportunities to compare patients’ subjective notions of their mental health with behavioral data to further investigate where these data are aligned and misaligned, charting a path toward more data-driven, measurement-based clinical decision-making. However, despite these promises, participants were simultaneously worried about how passively collected PGHD may change clinical workflows, in particular disrupting the norms surrounding how and when patient mental health information is gathered and used for clinical decision-making. In this discussion, we contextualize our results within the literature and attempt to reconcile these opportunities and challenges regarding the use of passively collected PGHD in clinical decision-making to best serve both the patient and their clinician. The implications suggested in this discussion are summarized in Textbox 3.

Our participants had mixed experiences with and opinions on the use of passive PGHD in clinical decision-making. Specifically, participating clinicians did not describe well-defined use cases for passive PGHD within current care pathways, instead describing how they currently reviewed passive data only when patients chose to bring these data into a clinical encounter. As Wu et al [26] echo, although passive PGHD can capture a wealth of information on patient behavior, it is less clear how this information adds value to improving patient care. Studies often focus on the technical efforts of collecting, deriving signals, and visualizing passive PGHD [12,40,41], but less attention has been paid to translational research showing the value of using PGHD in improving mental health care [42], and trials that have attempted to measure this value thus far have shown mixed results [43,44]. Thus, more evidence on how passive PGHD should be used for clinical decision-making may be required to further engage clinicians.

**Summary of themes with implications discussed.**

**Current passive patient-generated health data (PGHD) use is patient driven**

- Researchers can continue to build an evidence base showing specific use cases in which PGHD improved care and frameworks for evidence generation against these use cases.
- Before introducing passive PGHD, organizations should broadly understand clinicians’ familiarity with using PGHD and build clinical education programs describing how PGHD can be used for clinical decision-making.

**Active versus passive data as subjective versus objective data**

- Passive PGHD may offer an objective view into patient behavior and physiology, but clinicians perceive the information gained from passive PGHD as complementary to but not the same as mental health symptoms.
- In this context, passive PGHD could be used to give an outside opinion on symptoms, providing clinicians and their patients with contextual data on the patient from outside the clinic.
- Use of passive PGHD for clinical decision-making may be treatment specific or patient specific.

**Passive PGHD must be delivered at appropriate times for action**

- Passive PGHD gathering and use was found to disrupt clinical norms, organizational care pathways, and processes.
- Passive PGHD may be able to provide a near-continuous lens into the patient state, but the real-time nature of PGHD may overburden clinicians, who may already be experiencing burnout.
- Guidelines for passive PGHD use could borrow from existing practices in medicine, such as prescriptions and laboratory tests, to restrict use to moments when both patients and clinicians are interested in and have the bandwidth to use these data.

**Protecting patient privacy**

- Integrating patient goal setting into passive PGHD consent may increase patient engagement in treatment and ensure that PGHD use is in line with patients’ current goals within care.
- Designing new therapy practices that use passive PGHD would establish norms surrounding use in an evolved therapeutic frame.

PGHD researchers can draw lessons from other interdisciplinary collaborations, building an evidence base showing how data-driven technologies can improve clinical care [45]. For example, machine learning has been used for clinical decision support, documentation summarization, and aid in medical image–based diagnosis [46-49]. In each of these solutions, machine learning has supported progress toward clinical goals (eg, accurate diagnosis and prognosis) and improved clinician efficiency (eg, reduced image analysis time), illustrating the power of machine learning to create new capabilities and address clinical pain points while minimally disrupting clinical workflows. Analogously, mental health clinicians may only become interested in using passive PGHD by building an evidence base of specific clinical challenges that passive PGHD-based mental health tools have solved. Researchers can engage practitioners to uncover these clinical challenges and create instructions for how passive PGHD may solve these challenges, along with frameworks for evaluating efficacy within each specific use case to better support evidence generation. This work can coincide with ongoing efforts to better validate and standardize digital mental health measurements, often called *digital biomarkers* within the digital phenotyping paradigm, to detect condition-specific symptoms across patients, making the use of passive PGHD more feasible [30,50].

A promise of passive PGHD often cited in the literature is their ability to offer objective information on mental health in contrast to more traditional measures of mental health, in particular patient self-reports [16,51]. Although our results do not contest the objectivity of using digitally tracked data to measure behavior, they do question the objectivity of these data with respect to measuring a patient’s mental health. Researchers in human-computer interaction have echoed this point that digital measures may reduce mental health to biobehavioral data points, missing more interpretive aspects of mental health that cannot be easily quantified using passive tracking [20,33]. These critiques may also apply to specific forms of active PGHD, for example, behavioral data collected during health-related active tasks, although participants did not discuss these forms of active data during the interviews. However, our findings also show that prioritizing interpretive data alone can be problematic depending on the context. For example, as P4 highlighted, self-reported data collected from a patient experiencing mania should not be interpreted at face value. These tensions highlight the importance of contextualizing the patient’s condition when considering the use of both active and passive PGHD.

Our participants were interested in using passive PGHD to help patients further engage in their care, providing real-world behavioral data to understand treatment progression. As P11 and others mentioned, passive PGHD could create opportunities for patients and their clinicians to engage in measurement-based care, giving patients the ability to reflect on the efficacy of their treatment through actual behavior changes. Prior work reinforces providers’ interest in using passive tracking technologies to provide outside opinions on symptoms, giving patients opportunities to validate their progression through treatment using passively tracked data [27]. This perspective is considered in the broader literature on passive tracking and computational psychiatry, which suggests that passive PGHD could be a tool to reveal additional contextual information on behaviors outside the clinic, further explaining underlying disease mechanisms or changes in disease severity [15,52]. However, this perspective should not completely nullify the potential to use passive PGHD for remote symptom measurement, which motivates machine learning research investigating whether passive data can near-continuously predict self-reported mental health symptoms [53-55]. Considering the gap in receiving adequate psychiatric services when one experiences symptoms, passive PGHD symptom measurement may augment, not reduce, mental health measurement by remotely flagging those in need of a clinical follow-up, where more traditional, interpretive measures of mental health could then be administered [56,57]. Disentangling the different gaps in care that passive PGHD can fill will be essential for implementing these tools in a clinically useful way.

Participants in our study also expressed a variety of concerns regarding how PGHD might disrupt clinical norms, organizational care pathways, and processes. Specifically, participants described how the real-time nature of passively collected PGHD may overburden already burned out clinicians (P4), citing the need for actionable data only at opportune moments when clinicians have the bandwidth to care for a patient (P2). Existing data-gathering technologies used by clinicians, such as electronic health records, have created friction in clinical workflows by increasing administrative data entry tasks and induced “information overload” through saving a large volume of information, making it more difficult for clinicians to prioritize data needed for patient care [58]. Thus, organizations intending to use passive tracking technologies for clinical decision support are challenged to balance the promise of these tools for near-continuous, remote monitoring while fitting into clinical workflows that intentionally limit providers’ interactions with patients’ data to moments surrounding the clinical visit to reduce fatigue [59,60].

Thus, participants speculated on how passive PGHD could be used for clinical decision-making without exacerbating clinician fatigue. For example, P4 proposed dosing passive PGHD use similarly to a prescription, limited to a period in which it makes sense to hyperfocus on specific connections between behavior and health. Prescription models are often used to regulate the distribution of existing passive biometric data collection technologies, including continuous glucose monitors [61], and researchers have proposed “personalized activity prescriptions” for behavioral tracking, where clinicians and their patients can set specific health goals, which are tracked using passive activity monitors [62]. Continuing this analogy, these prescriptions, similar to many prescription medications, would have a beginning and end of use as well as specific guidance for appropriate use. This guidance may mediate participants’ concerns about collecting passive PGHD on a long-term basis and enabling patients’ obsessive tendencies toward self-tracking [27]. Another possibility raised by a participant would be to treat passive PGHD collection like a laboratory test, where clinicians request the data during a clinical encounter for a specific purpose, providing boundaries on when clinicians use PGHD. This PGHD lab would not make use of passive PGHD’s potential to provide near-continuous measurements [59]. However, thinking through how passive PGHD may fit within current clinical norms and practices (eg, prescriptions and laboratory tests) could create opportunities to augment future clinical workflows to realize the full potential of these near-continuous data sources.

Finally, participants highlighted patient privacy concerns regarding the collection and sharing of PGHD [62-64]. Patients living with mental health conditions may be particularly concerned about the perceived loss of privacy because of the societal stigma surrounding mental health disorders [65]. Simultaneously, establishing norms around data sharing is important in psychiatry; as our participants stated, specific types of treatments (eg, psychodynamic therapy) ask therapists to see the world through their patients’ eyes, often defined as the *therapeutic frame* [30,33,66]. As Nissenbaum [67] notes in their theory of privacy as *contextual integrity*, norms surrounding data sharing are tied to context (ie, in our case, the therapeutic frame). Thus, passive PGHD, which can provide a detailed window into patients’ lives outside the clinical encounter, may disrupt existing norms conceived by the therapeutic frame.

The concerns surrounding privacy highlight again the importance of patient engagement with their data for these data-driven interventions to be acceptable. To address privacy and data sharing concerns, P2 suggested forgoing simple consent procedures (eg, an “I agree” button) to instead work with patients to find opportunities to collect PGHD in alignment with patients’ treatment goals. For example, a patient may select exercise as a goal to motivate consent for the collection of step count or other activity data. In concordance with privacy scholars, such active, potentially recurring consent policies give patients opportunities to make personalized choices in their treatment plan as well as reflect on data-sharing practices [68]. By taking a proactive approach to patient privacy, providers can also create opportunities for collaborative goal setting, which may increase treatment engagement [69]. With regard to violating the therapeutic frame, new research efforts to measure the clinical actionability of traditionally collected collateral information (eg, conversations with other providers and family members) could be extended to digitally collected collateral information, including passive PGHD [30,70]. Through the judicious application of passive PGHD, clinicians can enhance the therapeutic relationship with their patients, reshaping the traditional concept of the therapeutic frame for a new digital age.

### Implications

Our findings have a variety of implications for researchers and practitioners regarding the adoption of technology-enabled, passively collected PGHD in clinical decision-making. First, our findings showed mixed mental health clinician experiences with passive PGHD. Thus, medical centers, should they endorse practices that use PGHD, may wish to invest in research and training programs to build and disseminate an evidence base showing the efficacy of using PGHD in mental health care. In addition, medical centers can invest in technological and organizational infrastructure to promote the effective uptake of passive PGHD-sharing programs. In addition, our interviews highlight that a primary benefit of passive PGHD is that they provide clinicians with contextual data from outside the clinic. However, the potential of passive PGHD to provide a near-continuous lens into the patient state may further contribute to physician burnout and violate patient privacy if proper safeguards are not enacted. Our findings suggest several methods to address these concerns, including prescribing or ordering PGHD similarly to a medication or laboratory test and creating conversations to reconsent patients into data-sharing programs. These ongoing conversations should address how the collection of PGHD aligns with patients’ specific treatment goals and how clinicians plan to use PGHD in combination with other types of information that patients share during clinical encounters.

### Limitations

This was a small-sample, qualitative semistructured interview study to elicit formative information regarding the use of passive PGHD for clinical decision-making. Although these methods allowed us to dive deeply with participants into a variety of tensions regarding the use of these tools, our results should not be considered generalizable to all mental health clinicians. We did not interview individuals in the broader mental health care and technology workforce (eg, social workers, nurses, home health aids, primary care physicians, and digital navigators), whose opinions will be extremely valuable when considering a future implementation of such tools. In addition, our small sample, composed mainly of psychiatrists, limits our ability to draw specific conclusions regarding different types of mental health clinicians (eg, more experienced, less experienced, schools of thought, and patient populations treated). We also conducted the interviews in early 2022, when mental health clinicians were dealing with the aftermath of the COVID-19 pandemic, which strained the mental health workforce. Thus, we were only able to reach and interview a limited number of clinicians, and we were cognizant of the amount of time we spent with individual clinicians to not strain our participants further.

### Future Work

Future work should seek to draw more generalizable conclusions for mental health clinicians. This could involve a larger interview study to qualify the experiences of a more diverse sample, along with a survey study to further quantify our findings and the status of mental health treatment. Future work can extend these results toward specific clinical practices and decision-making, showing whether and how passive PGHD are efficacious for both patients and their clinicians, determining how these data should be collected, delivered, and protected. Our results call for closing the research-to-practice gap for both PGHD and measurement-based psychiatric care as well as clarifying the information contained within passive PGHD and how this information complements more traditional forms of mental health data collected during clinical encounters. In addition, future work can create guidelines and clarify norms for best practices surrounding the use of passive PGHD for clinical decision-making. As our results show, this should include guidance for when passive PGHD may prove useful as well as consent procedures for enrollment and continued involvement in passive PGHD-sharing programs. Regulatory bodies and professional associations can produce clear, evidence-driven guidelines, which are imperative for maximizing the benefit of these novel data sources for both patients and their clinicians and ensuring that innovation serves real clinical needs and not just industry ambitions.

### Conclusions

We report a qualitative study to understand mental health clinicians’ perceptions and concerns regarding the use of technology-enabled, passively collected PGHD for clinical decision-making. Our findings highlight the need for building and disseminating an evidence base with guidelines for using technology-enabled, passively collected PGHD for clinical decision-making. This evidence base should clarify how to best use passive data alongside more traditional forms of active PGHD used for clinical decision-making, when clinicians should view passive PGHD to make treatment decisions, and how to best protect patient privacy within passive data–sharing programs. Academic medical centers and industry players can collaborate in clinical trials to generate clear evidence supporting the appropriate use of these novel tools for both patients and their clinicians, which would more effectively support their uptake and effective use in mental health care.

## Appendix

Multimedia Appendix 1 Interview guide.

Multimedia Appendix 2 Interview codebook.

## Abbreviations

PGHD: patient-generated health data
PRO: patient-reported outcome

## Data Availability

The data sets generated during and analyzed during this study are available from the corresponding author on reasonable request.
