# Supplementary material for: Understanding Mental Health Clinicians’ Perceptions and Concerns Regarding Using Passive Patient-Generated Health Data for Clinical Decision-Making: Qualitative Semistructured Interview Study
Source: JMIR Form Res. 2023 Aug 10;7:e47380. doi: 10.2196/47380 (PMC10450536; doi:10.2196/47380)
Supplement: Multimedia Appendix 1 [file formative_v7i1e47380_app1.docx]

# Multimedia Appendix 1 - Interview Guide

**Introduction**

Thank you for your participation today; we’re looking forward to our conversation. My name is [moderator’s name], and I will be moderating today’s discussion. This is [notetaker’s name], who will be taking notes during our conversation. We are researchers at Cornell University and Weill Cornell Medicine.

To start, I’m going to go over the overall goals of our study and remind you of your rights as a participant; then we’ll start the interview. We’re part of a research team interested in how mental health clinicians currently use patient-generated health data (PGHD) or may want to incorporate PGHD for clinical decision making. As part of that, today we’re going to ask you questions about PGHD and how you use or may wish to use PGHD in your practice. This session is estimated to take approximately 35 minutes. You may discontinue this interview at any time for any reason.

Do you have any questions before we begin?

**Introduction Questions**

1. Tell us about your work…
   1. [Probe] What types of psychiatric patients do you typically see?
   2. [Probe] What does a typical clinical encounter look like?

**Current understanding of PGHD in clinical decision making**

1. What is your current understanding of patient-generated health data (PGHD)?
   1. [If needed] Could you provide examples of PGHD you are familiar with?
2. If current understanding is limited:
   1. Based on your current understanding, are you interested in learning more about PGHD?
      1. Why or why not?
   2. Do you leverage any additional data sources outside of the traditional patient interview to support clinical decision making?
      1. If so, what are they?
   3. What avenues exist for you to learn about new tools to support clinical decision making?
      1. What are examples of recent tools you have learned about?
3. Have you ever thought about/used PGHD for clinical decision making? [Probe…]
   1. Why or why not?
   2. What would make PGHD ``trustworthy’’? In other words, what are your selection criteria in deciding which sources of PGHD to use (eg, clinical validation, cost, integration with current systems, clear understanding of relationship between PGHD and clinical constructs)?
4. [If the participant has used PGHD…]
   1. What types of PGHD have you used (eg, specific mobile apps, wearables, social media)?
   2. How do you currently learn about what sources of PGHD are available to use (eg, from colleagues, from patients, from professional societies)?
      1. Is there a better method that you would like to have to learn about PGHD?
   3. How do you factor in PGHD with other clinical data (eg, patient interviews and scales during appointment) to guide clinical decision making?

**Future interest in using PGHD for clinical decision making**

1. What types of PGHD (eg, web, mobile, wearable device) sound the most interesting to you?
   1. For example, qualitative measures like subjective reporting of mood, sleep quality?
   2. Quantitative measures like step counts, sleep duration, etc.?
2. How would you want PGHD to be presented to you (eg, self-reported by the patient, integrated within the EHR)?
   1. [Probe] How often would you use this PGHD?
      1. Only within the clinical-encounter?
      2. Would you want to look at it [the PGHD] outside of the clinical-encounter? Why or why not? [Potential privacy probes…]
3. What specific parts of the clinical-encounter do you think would most benefit from additional PGHD?

**Conclusion**

Thank you for taking the time to speak with us today.

1. Do you have any questions for us before we conclude the session?
2. Do you have any recommendations of other clinicians that you work with who may be interested in participating in this study?
