# Supplementary material for: Understanding Mental Health Clinicians’ Perceptions and Concerns Regarding Using Passive Patient-Generated Health Data for Clinical Decision-Making: Qualitative Semistructured Interview Study
Source: JMIR Form Res. 2023 Aug 10;7:e47380. doi: 10.2196/47380 (PMC10450536; doi:10.2196/47380)
Supplement: Multimedia Appendix 2 [file formative_v7i1e47380_app2.docx]

# Multimedia Appendix 2 - Interview Codebook

| **Theme**/Code |  |
| --- | --- |
| **Current Passive PGHD Use is Patient-Driven** | **Active versus Passive Data as Subjective versus Objective Data** |
| App-engagement data as PGHD  Behavioral data as PGHD  Data in "natural environment" as PGHD  PGHD supplemental to clinical data  Physiological data as PGHD | Collecting patient history  PGHD versus within visit data  Understanding interpersonal cues  Using clinical rating scales  Validity of passive measures  Using PGHD for medication management |
| **Passive PGHD Must be Delivered at Appropriate Times for Action** | **Protecting Patient Privacy** |
| Clinician training and using PGHD  Design and functionality of PGHD solutions  Integrating PGHD within the chart  Interpreting PGHD  Liability associated with PGHD  Measurement based care  Patient initiates PGHD usage  Patient receptivity to use PGHD  Patients obsessing over PGHD  PGHD and healthcare access  PGHD increasing patient engagement  "Prescribing" PGHD  Using PGHD in/around clinical encounter  Using PGHD within therapy sessions | Benefit outweigh privacy concerns  Clinician can continuously view PGHD  Consenting to sharing PGHD  Data as “collateral”  Data from patient’s home environment  Including family/friends in PGHD handover  Invasiveness of sharing PGHD  Patient disinterest in privacy implications  PGHD and the therapeutic frame |
